# Extracorporeal photopheresis in acute and chronic steroid‑refractory graft-versus-host disease: an evolving treatment landscape

**DOI:** 10.1038/s41375-022-01701-2

**Published:** 2022-09-24

**Authors:** Hildegard T. Greinix, Francis Ayuk, Robert Zeiser

**Affiliations:** 1 Division of Haematology, Medical University Graz, Auenbruggerplatz 38, 8036 Graz, Austria; 2 Department of Stem Cell Transplantation, University Medical Center Hamburg-Eppendorf, Hamburg, Germany; 3 Department of Medicine I - Medical Centre, Faculty of Medicine, University of Freiburg, Freiburg, Germany

**Keywords:** Drug development, Translational research

## Abstract

Patients with steroid-refractory graft-versus-host disease (GvHD) are known to have a poor prognosis and for decades no approved drug has been available to treat this serious condition. Although ruxolitinib, a selective Janus kinase (JAK)1/2 inhibitor demonstrated significantly higher response rates in randomized trials compared to the best available therapy, and thus, is of benefit in both acute as well as chronic GvHD, there is an urgent medical need to improve results, such as durability of responses, response in eye, liver and lung manifestations and reduction of infectious complications. In this “Review” article we would like to offer strategies for improving treatment results in patients with steroid-refractory GvHD by combining ruxolitinib with extracorporeal photopheresis (ECP), a leukapheresis-based immunomodulatory treatment frequently applied in T-cell mediated immune disease including GvHD. Our article explores key published evidence supporting the clinical efficacy of both ruxolitinib and ECP in the treatment of GvHD and highlights their potentially complementary mechanisms of action.

## Introduction

Graft-versus-host disease (GvHD) is a serious complication of allogeneic hematopoietic cell transplantation (HCT) and can be categorized into acute (aGvHD) and chronic (cGvHD) subtypes. Despite current prophylactic strategies, aGvHD can occur in up to 50% of patients including 14% with severe (grades 3–4) involvement that is associated with a mortality of 36% [1]. Major pathophysiological pathways that drive aGvHD have been described elsewhere [2–4].

Chronic GvHD remains the prevailing cause of nonrelapse mortality (NRM) in patients surviving longer than two years after HCT, negatively influencing both quality of life and long-term outcomes [5, 6]. It can inflict debilitating tissue injury due to fibrosis and puts patients at high risk for death from infections. The pathophysiology of cGvHD is more complex and involves multiple, distinct interactions among alloreactive and dysregulated T and B cells and innate immune populations, including macrophages, dendritic cells (DC) and neutrophils [5, 7–9]. Persistent immune dysregulation and alloreactivity are a hallmark of cGvHD and affected patients lack normal immune reconstitution [7, 9].

Corticosteroids are the first-line treatment of choice for both acute and cGvHD. Approximately 60% of patients with aGvHD do not respond to first-line therapy or recur after a response and are considered steroid-refractory (SR) with a six-month survival rate of around 50% and long-term survival rates of only 5–30% [10]. Extracorporeal photopheresis (ECP), mTOR inhibitors, and mycophenolate mofetil (MMF) were most frequently administered to patients with SR-GvHD. In May 2019 the FDA approved the Janus kinase (JAK) 1/2 inhibitor ruxolitinib for SR aGvHD in adult and pediatric patients 12 years and older based on an open-label, single-arm, multicenter trial including 49 patients with grades 2-4 SR aGvHD (REACH 1). In September 2021 FDA approval of ruxolitinib for SR cGvHD based on the results of the REACH3 study was obtained.

## Janus kinase inhibitors in acute and chronic GvHD

**Fig. 1:**
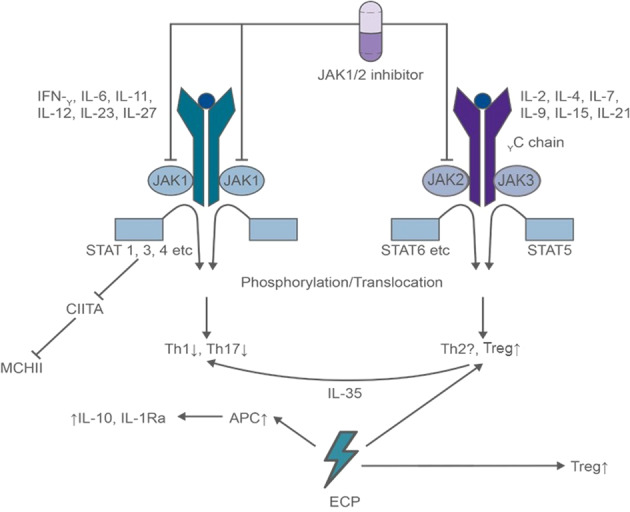
Complementary mechanisms of action of extracorporeal photopheresis plus ruxolitinib. Adapted from Teshima [79] by Costello Medical, UK. Proinflammatory cytokines, such as IFN-γ, IL-6, and IL-12, ignite serial phosphorylation of JAK1/2, cytokine-receptor chains, and STAT1/3/4. Phosphorylated STATs form heterodimers or homodimers, translocate to nucleus, resulting in transcriptions of Th1- or Th17-related genes. gC subunits of IL-2, IL-4, IL-7, IL-15, and IL-21 receptors are associated with JAK3 and STAT5 signaling. JAK1/2 selective inhibition spared the IL-2–JAK3–STAT5 signal and therefore may spare Tregs. Reinfusion of ECP-treated cells leads to phagocytosis by APCs, secretion of anti-inflammatory cytokines, modulation of T cells toward a Th2 phenotype and promotion of Treg cell generation. Abbreviations: JAK Janus kinase, IFN-γ interferon-gamma, IL interleukin, STAT Signal transducer and activator of transcription, Th T helper cells, APC antigen-presenting cells, Treg regulatory T cells, ECP extracorporeal photopheresis.

Mechanisms of action of the tyrosine kinase inhibitor that targets the JAKs 1 and 2 have been reviewed elsewhere [5, 11–14] and are shown in Fig. 1.

### JAK-inhibition in acute GvHD

In a multicenter, randomized, open-label, phase III trial including 309 patients with SR aGvHD in need of second-line treatment the efficacy and safety of oral ruxolitinib at 10 mg twice daily was compared to investigators’ choice consisting either of antithymocyte globulin (16%), etanercept (21%), ECP (31%), sirolimus, everolimus, infliximab (13%), methotrexate (MTX), MMF (22%) and mesenchymal stromal cells (13%), respectively [15]. Overall response rate (ORR) at day 28 was 62% in the ruxolitinib arm compared to 39% in the control arm (Odds ratio OR, 2.64, *p* < 0.001) and complete response (CR) rates were 28% and 20%. Durable overall response at day 56 was 40% in the ruxolitinib arm and thus, higher than in the control arm with 22% (OR 2.38, *p* < 0.001). The median failure-free survival (FFS) was considerably longer with ruxolitinib than with control (five months vs one month, Hazard ratio, HR 0.46), median overall survival (OS) was 11 months in the ruxolitinib group and 6.5 months in the control (HR, 0.83), respectively.

Since ruxolitinib administration can lead to cytopenias due to co-inhibiton of JAK2, specific JAK1 inhibitors were developed to reduce cytokine signaling without side effects [11]. Based on preclinical data [11], itacitinib, a highly selective JAK1 inhibitor was administered at 200 or 300 mg in a phase I study and induced an ORR of 78.6% and 66.7% [16]. GRAVITAS-301, an international, double-blind, adaptive (group sequential design) phase 3 study showed an ORR at day 28 of 74% including a CR rate of 53% for itacitinib and 66% (CR 40%) for placebo (OR for ORR 1.45, *p* = 0.078) [17]. The authors concluded that the observed improvement in ORR at day 28 with the addition of itacitinib versus placebo to corticosteroids did not reach the prespecified significance level, however the CR rate was significantly higher in patients with high-risk aGvHD.

Baricitinib, a best-in-class JAK1/2 inhibitor, inhibited interferon-gamma receptor and interleukin-6 receptor signaling, prevented GvHD with 100% survival and reversed ongoing GvHD in a fully HLA-mismatched preclinical model [18]. Baricitinib was superior to ruxolitinib in preclinical murine models regarding increases of Treg cells while decreasing helper T cell 1 and 2 (Th1 and Th2) cell differentiation and reducing the expression of MHC class II and costimulatory molecules CD80/86 on APCs. Clinical studies on the use of baricitinib in aGvHD are currently ongoing.

### JAK-inhibition in chronic GvHD

In the REACH3 study, a randomized, open-label, multicenter clinical trial of ruxolitinib compared to best available therapy (BAT) for SR cGvHD 329 patients received either ruxolitinib at 10 mg twice daily or BAT including ECP (35%), MMF (22%), MTX, rituximab, everolimus, sirolimus, infliximab, pentostatin, imatinib or ibrutinib (17%) [19]. ORR at week 24 according to NIH criteria [20] was 50% for the ruxolitinib arm and 26% for the BAT arm (*p* < 0.0001) including 7% and 3% CR rates. Ruxolitinib led to longer median FFS than control (>19 months vs 6 months, *p* < 0.001) and higher symptom response at week 24 according to the Lee symptom scale (24% vs 11%, *p* = 0.001). Among patients with a response at any time, the probability of maintaining a response at 12 months was 68.5% in the ruxolitinib arm compared with 40% in the BAT arm. Of note, ORR to ruxolitinib in eyes, liver, and lung were 26%, 24%, and 9% and thus, still unsatisfactory.

Organ responses and results of other prospective and retrospective studies on the use of ruxolitinib for SR acute and cGvHD are shown in Table 1a [15, 19, 21–32].

**Table 1:** **a** Summary of key evidence for ruxolitinib in treatment of steroid-refractory GvHD. **b**. Summary of key evidence for ECP in treatment of steroid-refractory GvHD.

Study	Trial design	Treatment	ORR %/CR%	Other results
**a**				
Acute GvHD				
Jagasia et al. [21] (*n* = 71)	Open-label, single-arm, multicentre phase II	Ruxolitinib + CS	55/27	ORR in skin, liver, and gut 61%, 27%, and 46%; median DOR at 6 mo was 345 days; 6-mo and 12-mo OS 51% and 43%; 6-mo and 12-mo NRM 44% and 53%
Zeiser et al. [15] (*n* = 309; 154 vs155)	Open-label, randomized, multicentre phase III	Ruxolitinib + CS vs best available therapy	62 vs 39 (*p* < 0.001)/ 34 vs 19 ns	ORR (Day 56): 40% vs 22% (*p* < 0.001); median FFS 5 mo vs 1 mo: median OS 11 mo vs 6.5 mo; 6-mo NRM 36% vs 43%; 12-mo NRM 43% vs 45%; CMV infection 26% vs 21%.
Moiseev et al. [22] (*n* = 32, children and adults)	Prospective	Ruxolitinib + CS± other IS	75/63	Reduced ORR in grade III-IV aGvHD, liver GvHD severity, grade IV GI GvHD; OS 59%; CMV infection 59%
Liu et al. [23] (*n* = 40, haplo)	Prospective	Ruxolitinib + CS	85/62.5	Worse ORR and OS in liver involvement; ORR in skin, liver, and gut 83%, 48%, and 82%; 6-mo OS and TRM 57% and 33%; GvHD recurrence 26.5%
Zeiser et al. [24] (*n* = 54)	Retrospective	Ruxolitinib + CS + IS	81.5/46	Median time to response 1.5 weeks; 6-mo OS 79%, aGvHD recurrence in 7%; CMV infection in 33%
Leung et al. [25] (*n* = 26)	Retrospective	Ruxolitinib + CS	86/36	CR at 3 mo 68%; 1-yr and 2-yr OS 58% and 37%
Wei et al. [26] (*n* = 23)	Retrospective	Ruxolitinib + CS ± IS	86.9/56.5	ORR in skin, liver and gut 88%, 67% and 92%; 1-yr and 2-yr OS 83% and 75%; CMV reactivation in 52%
Gomez et al. [27] (*n* = 23)	Retrospective	Ruxolitinib + CS	69.5/21.7	ORR in skin, liver, and gut 69%, 69%, and 67%; CR in skin, liver, and gut 19%, 23%, and 19%; 6-mo OS 47%
Chronic GvHD				
Zeiser et al. [19] (*n* = 329;165 vs 164)	Open-label, randomized, multicentre phase III	Ruxolitinib + CS vs best available therapy + CS	50/7 vs 26/3	ORR in skin 41%, mouth 50%, upper GI 50%, lower GI 53%, joints 38%; median FFS at week 24 > 18.6 mo vs 5.7 mo (*p* < 0.001); 6-mo FFS 75% vs 44.5%; symptom release at week 24 24% vs 11% (*p* = 0.0001); 1-yr OS 81% vs 84%
Moiseev et al. [22] (*n* = 43, adults and children)	Prospective	Ruxolitinib + CS ± other IS	81/21	No changes in eye, lung, joint, and genitalia severity; OS 85%; NRM 7%
Wang et al. [28] (*n* = 70)	Retrospective	Ruxolitinib + CS ± other IS	74/49	Lower ORR in severe cGvHD; best ORR in mouth with 83%; lowest ORR in liver with 64%; 1-yr OS 66%; 1-yr NRM 20%
Gomez et al. [27] (*n* = 56)	Retrospective multicentre	Ruxolitinib + CS	57/3.5	ORR in skin sclerosis 56%, in lung 61.5% and gut 56%; 1-yr OS 81%; CMV infection in 20%
Yang et al. [29] (*n* = 53 children)	Retrospective	Ruxolitinib + CS	81/28	GvHD recurrence in 31%; 6-mo OS and EFS 100% and 97%
Redondo et al. [30] (*n* = 48)	Retrospective	Ruxolitinib + CS	77/15	ORR of 77% in sclerotic skin, 45% in gut, and 33% in lung disease; similar ORR in moderate and severe cGvHD of 74% and 77%; 2-yr NRM 15%; 2-yr OS 83%
Modi et al. [31] (*n* = 46)	Retrospective	Ruxolitinib + CS ± other IS	48/10	12-mo ORR of skin 25%, 60% mouth, 26% eye, 10% lung and 41% joints; 1-yr FFS 54%
Zeiser et al. [24] (*n* = 41)	Retrospective, multicentre	Ruxolitinib + CS ± other IS	85/7	6-mo OS 97%; CMV infection in 15%
Xue et al. [32] (*n* = 36)	Retrospective	Ruxolitinib + CS	62/na	Significant improvement in skin, genital and oral GvHD at 3 mo, ocular GvHD at 6 mo; 1-and 2-yr OS 81% and 74%; 1-and 2-yr TRM 19% and 19%
Wei et al. [26] (*n* = 32)	Retrospective	Ruxolitinib + CS ± other IS	78/25	ORR for mouth 91%, eye 80%, liver 80%, lung 44%: lower ORR in severe cGvHD of 65%; 1-and 2-yr OS 81% and 73%
Leung et al. [25] (*n* = 31)	Retrospective	Ruxolitinib + CS	86/17	6-mo ORR and CR 83% and 52%; 1-and 2-yr OS 94% and 81%
**b**				
Acute GvHD				
Greinix et al . [34] (*n* = 59)	Prospective, single-arm, phase II	ECP + CS in second-line	70/60	CR in skin 82%, liver 61% and gut 61%; 4-yr OS 47% (59% and 11% in ECP responders and non-responders); 4-yr TRM 36% (14% and 73% in ECP responders and non-responders)
Amat et al. [35] (*n* = 37)	Prospective, multicenter	ECP + CS in second-line	73/40.5	ORR in skin 71%, liver 54.5% and gut 67%; significant longer OS in CR pts (median > 47 mo vs 12 mo)
Jagasia et al. [36] (*n* = 108)	Retrospective, multicentre	ECP + CS vs Inolimomab/Etanercept + CS in second-line	66/54 vs 32/20 (*p* = 0.001)	ECP as independent predictor of ORR (HR, 3.42, *p* = 0.007), OS (HR, 2.12, *p* = 0.018); ECP associated with superior OS (HR, 4.6, *p* = 0.016) in SR aGvHD grade II and lower NRM (HR, 0.45, *p* = 0.018)
DasGupta et al. [37] (*n* = 128)	Retrospective, multicentre	ECP + CS in second-line	77/67	6-mo-FFTF 77%; 2-yr OS 56%; 2-yr TRM 34%
Worel et al. [38] (*n* = 99)	Retrospective, single center	ECP + CS in second-line	75/53	ORR in skin 80%, liver 61% and GI 75%; 1-yr and 5-yr TRM 22% and 31%; 1-yr and 5-yr OS 69% and 50%
Calore et al. [39] (*n* = 72, children)	Retrospective	ECP + CS ± other IS	83/64	CR in skin in 70%, liver in 84%, and gut in 71%; 5-yr OS 71%
Niittyvuopio et al. [40] (*n* = 52)	Retrospective, single center	ECP + CS in second-and third line	62/48	CR in skin 77%, liver 33%, and gut 34%; 1-yr OS 51%
Perotti et al. [41] (*n* = 50, children)	Retrospective, single center	ECP + CS	68/32	ORR in skin 83%, liver 67%, and gut 73%; 1-yr OS 64%
Malagola et al. [42] (*n* = 45)	Retrospective, multicentre	ECP + CS in second-line	Na/91	CR in grade II 97% and grades III/IV 67%
Messina et al. [43] (*n* = 33, children)	Retrospective, single center	ECP + CS ± other IS	75/54	CR in skin 76%, liver 60% and gut 75%; 5-yr OS 69%
Chronic GvHD				
Flowers et al. [44] (*n* = 95; 48 vs 47)	Prospective, randomized, multicenter	ECP + CS ± other IS vs CS ± other IS	40 vs 10 at w12 in skin (*p* = 0.002)	ORR in eye 30% vs 7% (*p* = 0.04) and mouth 53% vs 27% (*p* = 0.06); median % improvement of TSS at week 12 14.5% vs 8.5%, at week 24 31.4% in the ECP arm.
Greinix et al. [45] (*n* = 29)	Prospective, crossover, multicentre	ECP + CS ± other IS	31% at w24 in skin	ORR in liver 50%, mouth 70%, and joints 36%; median % improvement of TSS at week 24 25.8%
Sakellari et al. [46] (*n* = 88)	Prospective, single center	ECP + CS	73/40	ORR in skin sclerosis 83%, visceral involvement 53% and lung 27%; 5-yr TRM 24%; 5-yr OS 64.5%
Gandelman et al. [47] (*n* = 77)	Prospective, multicentre	ECP + CS ± other IS	62/14	ORR in skin 55%; ECP responses independent of risk factors of poor outcome
Dignan et al. [48] (*n* = 82)	Retrospective, single center	ECP + CS ± other IS	79/na	ORR in skin 92% and mouth 91% at 6 mo; 3-yr OS 69%
Couriel et al. [49] (*n* = 71)	Retrospective, single center	ECP + CS ± other IS	61/20	ORR in skin 57%, liver 71% and mouth 78%; 1-yr OS 53%; response to ECP and platelet count at ECP start significantly predict NRM
Greinix et al. [50] (*n* = 47)	Retrospective, single center	ECP + CS ± other IS	83/na	CR in skin 68%, mouth 81%, and liver 68%

*ORR* overall response rate, *CR* complete response rate, *CS* corticosteroids, *DOR* duration of response, *Mo* months, *OS* overall survival, *NRM* nonrelapse mortality, *Ns* not significant, *FFS* failure-free survival, *CMV* cytomegalovirus, *IS* immunosuppressants, *aGvHD* acute graft-versus-host disease, *GI* gastrointestinal, *Yr* year *cGvHD* chronic graft-versus-host disease *EFS* event-free survival, *TRM* transplant-related mortality, *Na* not applicable, *ECP* extracorporeal photopheresis, *ORR* overall response rate, *Pts* patients, *HR* hazard ratio, *SR* steroid-refractor, *FFTF* freedom from treatment failure, *TSS* total skin score.

## Extracorporeal photopheresis

Besides ruxolitinib ECP has been widely used for treatment of GvHD [33]. ECP, a cell-based immunotherapy that involves the reinfusion of autologous mononuclear cells after exposure to 8­methoxypsoralen and ultraviolet A (UVA) light irradiation, is an established, clinically effective second-line therapy for SR acute and cGvHD (Table 1b) [34–50]. ECP’s immunomodulatory effect is antigen specific and reinfusion of apoptotic cells due to UVA irradiation leads to phagocytosis by nonexposed APCs, secretion of anti-inflammatory cytokines and chemokines, modulation of T cells toward a Th2 phenotype, maturation of DCs and promotion of Treg cell generation (Fig. 1) [51].

### ECP in acute GvHD

In a MHC minor mismatch mouse model of aGvHD the transfer of cells treated ex vivo with ECP significantly reduced established severe aGvHD, suppressed allogeneic responses of donor effector T cells that had never been exposed to psoralen and UVA radiation and increased the number of Treg cells derived from both the donor T-cell and bone marrow (BM) grafts [52]. Murine models showed that alloreactive apoptotic T cells are essential for the induction of ECP-mediated tolerance since ECP-treated cells from healthy mice with BM donor’s genetic background were not effective in ameliorating aGvHD in a MHC I and II mismatched mouse model [53].

Mice receiving ECP-treated cells demonstrated improved immune reconstitution, which is consistent with reduced aGvHD and with the relative immunocompetence of patients who receive ECP therapy compared with immunosuppressive treatment [54, 55]. During ECP treatment of both acute and cGvHD patients’ quantity and quality of antiviral and antileukemic effector cells were preserved and the frequency of Foxp3^+^CD4^+^ Treg cells and CD24^+^CD38^high^ regulatory B cells was considerably increased in aGvHD patients [55].

In prospective studies on SR aGvHD ORR of 69%, ORR in skin of 84%, liver of 55%, and gut of 65% have been reported [56]. Jagasia and colleagues compared ECP with anti-cytokine therapy as second-line treatment for SR aGvHD in a retrospective analysis reporting ORR of 66% and 32% in the ECP and the anti-cytokine cohort including CR rates of 54% and 20% [36]. In multivariate analysis, ECP was an independent predictor of response (HR 3.42, *p* = 0.007) and OS (HR 2.12, *p* = 0.018). The administration of ECP was associated with lower NRM (HR 0.45, *p* = 0.018) and in patients with SR aGvHD grade II with superior OS rates (HR 4.6, *p* = 0.016).

### ECP in chronic GvHD

In a multicentre, randomized study comparing ECP and conventional immunosuppressive treatment (IST) with conventional IST alone including 95 patients with cGvHD after a median duration of 50 and 55 weeks of prestudy steroid treatment the ORR at week 12 in skin was 40% in the ECP arm compared to 10% in the control arm (*p* = 0.002) [44]. Best ORRs were observed in oral mucosal involvement with 53% in the ECP arm and 27% in the control arm, respectively. At week 12, the median targeted symptom assessment scores improved in the ECP arm by 19% compared with 2.5% in the control arm (*p* = 0.01). Since ECP does not induce general immunosuppression [54], risk of infections compared with other IST is not increased. In the randomized, multicentre study infections were observed in 18% of patients in the ECP arm and 16% in the control arm, respectively [44]. ECP has a steroid-sparing effect as reported by multiple investigators of prospective and retrospective studies [36, 44–48]. In the randomized study by week 12 at least a 50% reduction in steroid dose and a daily steroid dose below 10 mg were achieved in 21% of patients in the ECP cohort and 6% of the control (*p* = 0.04) [44]. In an open-label crossover ECP study in 29 patients with lack of improvement or progression of SR cGvHD under 12 weeks of conventional IST patients served as their own controls [45]. Significantly more patients in the ECP study compared with the initial non-ECP period achieved a CR or partial response (PR) of the skin (26% vs 8%, *p* = 0.04), oral mucosa (65% vs 27%, *p* = 0.009) and ocular involvement (27% vs 7%, *p* = 0.04) at week 12 after crossing over to ECP treatment. ECP was generally tolerated well but patients need a reliable venous access for prolonged periods of time that can require central venous catheters and the infectious risk associated.

The prospectively randomized studies on the use of ruxolitinib or ECP in SR cGvHD cannot be compared well since important changes such as staging and response assessment of cGvHD according to NIH criteria [20, 57], primary endpoints (ORR at week 24 in REACH3 vs median percent change in total skin score at week 12 in the ECP study), and patient-reported outcome measures included in response assessment meantime changed. Furthermore, REACH3 enrolled only patients in need of second-line therapy of SR cGvHD and not more advanced ones and ruxolitinib was the only systemic intervention allowed at enrollment whereas in the ECP study patients could also receive MMF and had a longer duration of cGvHD prior to study enrollment, respectively.

### Combination therapies

Patients with SR aGvHD that includes severe gastrointestinal (GI) involvement are in urgent need of efficient and safe salvage treatment to reduce their risk for infectious complications and organ toxicities. The ultimate goal to improve these patients’ prognosis is to rapidly achieve durable CR rates without disease flare-ups under steroid taper.

In a recently published single-center experience of combining ruxolitinib with ECP in 18 patients with severe SR aGvHD of lower GI-tract (50% each overall grades III and IV) and other organ manifestations (skin *n* = 7, liver *n* = 6, upper GI-tract *n* = 2), the majority of patients (*n* = 15, 83%) received ruxolitinib a median of 20 days before starting ECP [58]. Modemann and colleagues observed a best ORR of 55% including a CR rate of 44% and an additional PR rate of 11%, respectively. The mean daily steroid dose was 130 mg at diagnosis of SR aGvHD and at start of lead-in ruxolitinib (83% of patients) or ruxolitinib with ECP (17% of patients) treatment and could be tapered to less than 20 mg by day 21 and stopped after a median of 27 days. Although the authors did not present detailed data on the respective tapering of steroids under ruxolitinib alone or the combination with ECP, their results demonstrate the feasibility of a rapid steroid taper and discontinuation of steroids with this combinational strategy. Responding patients had a two-year OS of 70% with a median survival of 18 months.

In contrast to the REACH2 study that reported adverse events up to day 28 after start of ruxolitinib, Modemann and colleagues described maximum of worsening of cytopenia for the whole treatment course [15, 58]. Although not comparable due to different time periods assessed we would like to mention the incidences of cytopenias in both studies since these were the main therapeutic side effects. In the ruxolitinib arm of the REACH2 study anemia of any grade and grade ≥3 were seen in 30% and 22% whereas in combinational treatment of ruxolitinib with ECP 28% developed worsening of anemia about 1 to 2 CTC grades. Any grade and grade ≥3 thrombocytopenia occurred in 33% and 27% in the REACH2 study compared to 39% worsening of the platelet count about 1 to 3 CTC grades in combinational therapy. Main differences between these two studies were seen regarding leukopenia with 9% and 7% decrease of white blood cell count (WBC) in REACH2 and 50% worsening of leukocyte count in ruxolitinib with ECP, respectively. Cytomegalovirus (CMV) infection of any grade was observed in 26% in the REACH2 study whereas Modemann and colleagues reported 67% during ruxolitinib therapy prior to ECP. Eight patients (44%) had to stop either ruxolitinib or ECP due to cytopenia including 3 recovering platelet, hemoglobin, and leukocyte levels within 4 weeks.

In a retrospective analysis 23 patients with SR cGvHD (57% NIH grade 3, 91% beyond second-line treatment, and 87% with more than one organ involved), received the combination of ruxolitinib at 5–10 mg bid and ECP with two treatments on consecutive days every two to four weeks [59]. Thirty-five percent of patients started ECP and ruxolitinib treatment simultaneously, whereas 30% started ECP first and the median time of ECP therapy prior to combination treatment was 3.25 (1–7) months. During ECP alone the best response was PR in 43% (3/7) of patients and 57% (4/7) were non-responders. Thirty-five percent of patients started ruxolitinib treatment first a median of 15 (1–29) months prior to combination treatment. The best ORR to ruxolitinib alone was PR in 62.5% (5/8) and 37.5% (3/8) did not respond. Best ORR of ECP combined with ruxolitinib was 74% (17/23) including 9% CR and 65% PR and a two-year OS of 75% [59]. Thus, combinational treatment increased ORR in heavily pretreated patients with multiorgan involvement SR cGvHD and was able to improve outcome of patients after inadequate responses to ECP or ruxolitinib monotherapy. Patients received a median of six months of combination therapy.

In combinational therapy cytopenia occurred in 48% (11/23) of patients including 6 with preexisting cytopenia before ruxolitinib administration and thus, 22% (5/23) developed new cytopenia. Furthermore, 6 of 8 patients that had received ruxolitinib monotherapy first had cytopenia under ruxolitinib including 4 with resolution of cytopenia under combinational treatment. This is in contrast to the REACH3 study’s ruxolitinib arm where any grade and grade ≥3 anemia were observed in 29.1% and 12.7%, any grade and grade ≥3 thrombocytopenia in 21.2% and 15.2%, and any grade and grade >3 neutropenia in 10.9% and 8.5%, respectively [19]. However, due to small study size of the ruxolitinib monotherapy cohort in this retrospective analysis, no firm conclusions should be drawn from these results. Interestingly, only 2 patients had to stop ECP due to poor venous access (*n* = 1) and poor general condition most likely unrelated to ECP (*n* = 1). CMV reactivation was observed in 26% of patients given ruxolitinib and ECP compared to 5.5% in the REACH3 study. Serum levels of soluble interleukin-2 receptor started to decline with ruxolitinib monotherapy and further declined during combination treatment and thus, correlated with response.

In summary, the most valuable findings regarding side effects of combination therapy seem to be in the context of SR aGvHD the discontinuation of either ruxolitinib or ECP due to cytopenia in 44% of patients, the development of new cytopenia in 22% in the context of SR cGvHD, and the discontinuation of ECP due to poor venous access in only 1 of 23 patients. Due to the small patient number and the heterogeneity of start of therapies, these promising results should be tested prospectively in a patient cohort given ECP and ruxolitinib simultaneously from the beginning of treatment. Information on red blood cell and platelet transfusional support should be a secondary endpoint in any of these studies. Furthermore, addition of ECP in case of inadequate response to ruxolitinib monotherapy could be considered for another prospective study.

ECP monotherapy for 8 weeks followed by combination treatment with low-dose interleukin-2 (IL-2) for another 8 weeks was explored in a prospective phase II study in 25 patients with SR cGvHD who had a median of 3 (2–5) sites of organ involvement and a median of 2 (1–4) prior IS therapies [60]. ORR was 29% at 8 weeks after ECP alone and 62% at 16 weeks after combination treatment which appears comparable to that reported with low-dose IL-2 monotherapy [61]. ECP monotherapy led to a significant decline of CD4^+^ and CD8^+^ T cells with minimal changes of Treg and NK cells whereas the combination of ECP and IL-2 led to increases in Tregs and NK cells, respectively.

## Remaining challenges in treatment of GvHD patients

In a recent thorough review of the literature ruxolitinib (two studies) and ECP (five studies) were considered to be superior to other second-line therapies in patients with SR aGvHD due to better than expected six-month survival [62]. Of note, a preference for ECP instead of ruxolitinib use in patients with active infection or severe neutropenia or thrombocytopenia was expressed by the author [62]. This conclusion was based on a meta-analysis in patients with myeloproliferative neoplasms suggesting a clinically relevant incidence of opportunistic and viral infections in patients treated with ruxolitinib [63]. In the REACH2 study infections of grade 3 severity up to day 28 occurred in 34 patients (22%) in the ruxolitinib arm and in 28 patients (19%) in the control arm, respectively [15]. Among patients with infection, the median time to first infection of grade 3 severity was 0.8 months with ruxolitinib compared with 0.7 months with control therapy. In the REACH3 trial infections of any type occurred in 63.6% of patients given ruxolitinib compared with 56.3% who received control therapy including grade 3 infections in 19.4% vs. 18.4%. Viral infections were the most common with 33.9% and 29.1% in the ruxolitinib and control groups, followed by bacterial with 27.9% and 25.9% and fungal infections with 11.5% and 5.7%, respectively. This higher incidence of fungal infections in the ruxolitinib arm is reminescent of the occurrence of opportunistic infections during this treatment in patients with myelofibrosis [64]. Of note, CMV infection was similar in the REACH3 groups with 5.5% and 8.2%.

### Treatment of GvHD beyond ECP and ruxolitinib

Although the availability of FDA-approved novel drugs for SR-GvHD has expanded treatment options for a substantial number of patients, progression to irreversible fibrotic sequelae still occurs in many of them. Moreover, medication side effects, and persistence of immune dysfunction with serious infectious complications for prolonged periods of time, remain serious co-morbidities. Of note, median time to permanent discontinuation of immunosuppression in cGvHD patients was 69 months as recently reported [65]. Even in the REACH3 study most responses were partial ones and further improvement in ORR in organs such as eye, liver, and lung are warranted. Furthermore, patients in the REACH3 study had not prior been exposed to recently FDA-approved drugs such as ibrutinib and belumosudil whereas in the pivotal studies leading to FDA approval of both of these agents only 1 patient in the KD025 study with belumosudil had prior treatment with ruxolitinib allowing the assumption that cGvHD patients refractory to steroids and one of these novel agents could benefit from the other approved treatments available.

Based on preclinical data ibrutinib, an oral selective and irreversible inhibitor of Bruton’s tyrosine kinase in B cells and interleukin-2-inducible T-cell kinase in T cells, was investigated at an oral dose of 420 mg daily in an open phase Ib/II study with 42 patients (11 (26%) after prior ECP/PUVA) with active cGvHD with inadequate response to steroid-containing therapies [66]. In contrast to the REACH3 study with the primary endpoint of ORR at week 24, in the ibrutinib study NIH-defined best ORR at any time was evaluated [20, 67]. After a median follow-up of 13.9 months, best ORR was 67% and 71% of responders showed a sustained response for ≥20 weeks. With ibrutinib best organ responses were observed in skin (88%), mouth (88%), and GI (91%) whereas in the ruxolitinib arm in the REACH3 study ORR at week 24 were 41.2% in skin, 50% in mouth and 53.3% in lower GI manifestations [19]. Limitations of the ibrutinib study are the predominance of cutaneous and oral mucosal involvement in enrolled patients with cGvHD, with no data available on other important organ manifestations such as eyes, lungs, or joints/fascia and the lack of separation of cutaneous involvement into inflammatory and fibrotic features, leaving it unclear whether both responded equally well to ibrutinib. Favorable clinician-reported cGvHD efficacy results were complemented by results from patient-reported outcomes (PRO) data supporting the FDA’s positive benefit-risk assessment [68]. After a median follow-up of 26 months best ORR was 69% with 13 patients (31%) achieving a CR and 16 (38%) a PR [69]. Sustained responses of ≥20, ≥32, and ≥44 weeks were seen in 20 (69%), 18 (62%), and 16 (55%) of the 29 responders, respectively. Of note, the best ORR up to week 24 in the ruxolitinib arm of the REACH3 study was 76.4% and among responders the probability of maintaining a response at 12 months was 68.5% [19].

The most common adverse events of ibrutinib were fatigue (57%), diarrhea (37%), muscle spasms (28%), nausea (26%), and bruising (24%), all mostly grades 1 and 2 [66]. Infectious complications were reported for 69% of patients, including 36% grade ≥3 events and 2 infectious deaths while receiving ibrutinib. In 33% of patients, adverse events led to treatment discontinuation after a median of 1.8 months.

In July 2021, the FDA-approved belumosudil, an oral selective inhibitor of Rho-associated coiled-coil-containing protein kinase 2 (ROCK2), for patients 12 years and older with cGvHD after failure of at least 2 prior lines of systemic therapy based on the results of the KDO25 study with 65 patients including 71% with severe cGvHD and 48% with ≥4 organs involved [70, 71]. Five patients (8%) had prior ECP treatment and 20 patients (31%) ruxolitinib. ORR was 75% and responses were achieved across key subgroups with ORRs of 76% in patients with severe cGvHD, 67% in patients who had lung involvement and 77% in patients with >4 organs involved. Median duration of responses was 1.9 months and the median time from first response to death or new systemic therapy, was not reached. Responses were associated with quality-of-life improvements and steroid dose reductions. In a recently published randomized multicenter registration study evaluating belumosudil 200 mg daily (*n* = 66) and 200 mg twice daily (*n* = 66) in patients with cGvHD after 2–5 prior lines of therapy best ORR after a median follow-up of 14 months was 74% and 77% and median duration of response was 54 weeks [72].

Whereas some organ responses compare well between belumosudil and ruxolitinib such as skin (37% vs 41.2%), mouth (55% vs 50%) and esophagus (45% vs 50%), others are in favor of belumosudil such as eyes (42% vs 26%), lung (26% vs 8.6%), liver (39% vs 24.4%), joint/fascia (71% vs 37.8%) and lower GI (69% vs 53.3%), respectively. However, small patient numbers in both studies do not allow definite conclusions. Symptom reduction with belumosudil 200 mg daily and 200 mg twice daily was reported in 59% and 62% of patients, respectively. Also the adverse events of belumosudil are quite different with mainly fatigue (38%), diarrhea (33%), nausea (31%), and cough (28%). Grade ≥3 adverse events consisted of increases in liver enzymes (AST 10%, ALT 8%), pneumonia (8%), hypertension (6%), and hyperglycemia (5%). In the KD025 study grade ≥3 cytopenias were reported in 2 patients (4%) and no cases of CMV infection occurred [71].

During the last years more detailed insights into the pathophysiology of GvHD and thus, drugable targets obtained from solid preclinical data led to exploration of other agents such as abatacept in clinical trials. All these research activities will hopefully increase the therapeutic options for patients with SR-GvHD. Furthermore, select biomarkers or panels of biomarkers could guide the selection of specific therapeutic agents and be used to monitor responses to treatment [73]. Biomarkers predicting the potential for response to therapy are highly warranted but so far, limited data is available [73, 74]. In relation to ECP, robust biomarkers of GvHD would be highly useful in informing patient selection, intensity, and duration of ECP schedule, monitoring of response and other treatment decisions alongside the concurrent administration of other GvHD therapies. Few soluble and cellular biomarkers such as B-cell activating factor (BAFF), CD19^+^CD21^-^ B lymphocytes, and CD56^bright^ natural killer (NK) cells, so far, have been investigated prior to and during ECP therapy [75, 76]. In the REACH3 study evaluated blood-based biomarkers did not change over time and were not predictive of response to ruxolitinib or best available therapy with the possible exception of Reg3α for patients with GI involvement [77]. Thus, further studies are warranted assessing biomarker profiles prospectively regarding their potential for prediction of response to select novel therapies.

## Conclusions

In order to improve outcome of patients with SR-GvHD further, either more effective novel monotherapies could be investigated or therapeutic combinations with non-overlapping toxicities could be selected to increase efficacy and safety. Due to the excellent safety profile of ECP and its lacking interaction with other drugs ECP could be considered in combination with ruxolitinib in adults with SR-GvHD with the aim to improve CR rates and durability of response, reduce time under high dose corticosteroids leading eventually to reduced NRM and improved OS of patients afflicted. Considering the observed hematotoxicity of ruxolitinib with a higher incidence of ≥ grade III thrombocytopenia and anemia combination therapy might allow continuation of ECP during ruxolitinib pause and further reduction in steroid doses. Durability of responses and side effects of immunosuppression including relapse of malignant disease, organ toxicities, and severe infections have remained challenging in patients with SR-GvHD. No impairment of the antiviral and antileukemic immune responses during and after administration of ECP have been reported [55]. Therefore, prospective studies with this combination are highly encouraged. Ultimately, new insights are needed into how clinical manifestations or phenotypes of cGvHD are related to pathophysiologic mechanisms that are potential targets of novel therapeutic agents [78]. This could allow for more biologically relevant and individualized treatment approaches.

## Data Availability

The data that support this review are available from the corresponding author upon reasonable request.
